# Case study on climate change effects and food security in Southeast Asia

**DOI:** 10.1038/s41598-024-65140-y

**Published:** 2024-07-12

**Authors:** Daria Taniushkina, Aleksander Lukashevich, Valeriy Shevchenko, Ilya S. Belalov, Nazar Sotiriadi, Veronica Narozhnaia, Kirill Kovalev, Alexander Krenke, Nikita Lazarichev, Alexander Bulkin, Yury Maximov

**Affiliations:** 1 Skolkovo Institute of Science and Technology, Moscow, Russia; 2 FRC Biotechnology, Russian Academy of Sciences, Moscow, Russia; 3 Credit Risks Department, PJSC Sber, Moscow, Russia; 4 Institute of Geography, Russian Academy of Sciences, Moscow, Russia; 5 Research Center Interdata, Timertau, Kazakhstan; 6 Institute for Artificial Intelligence, Moscow State University, Moscow, Russia; 7 International Center for Corporate Data Analysis, Astana, Kazakhstan; 8 Theoretical Division, Los Alamos National Laboratory, Los Alamos, NM USA

**Keywords:** Environmental health, Computational science, Projection and prediction

## Abstract

Agriculture, a cornerstone of human civilization, faces rising challenges from climate change, resource limitations, and stagnating yields. Precise crop production forecasts are crucial for shaping trade policies, development strategies, and humanitarian initiatives. This study introduces a comprehensive machine learning framework designed to predict crop production. We leverage CMIP5 climate projections under a moderate carbon emission scenario to evaluate the future suitability of agricultural lands and incorporate climatic data, historical agricultural trends, and fertilizer usage to project yield changes. Our integrated approach forecasts significant regional variations in crop production across Southeast Asia by 2028, identifying potential cropland utilization. Specifically, the cropland area in Indonesia, Malaysia, Philippines, and Viet Nam is projected to decline by more than 10% if no action is taken, and there is potential to mitigate that loss. Moreover, rice production is projected to decline by 19% in Viet Nam and 7% in Thailand, while the Philippines may see a 5% increase compared to 2021 levels. Our findings underscore the critical impacts of climate change and human activities on agricultural productivity, offering essential insights for policy-making and fostering international cooperation.

## Introduction

Climate change has a substantial impact on crop production, which poses risks to food security globally^1^. The Secretary-General of the United Nations has highlighted that Least Developed Countries are particularly vulnerable to these risks, especially given rising food and energy costs^2^. Despite technological advances since the Industrial and Green Revolutions, climate change and weather variability remain the primary factors affecting crop production^3^. Anthropogenic factors exacerbate temperature and precipitation extremes, further compounding the issue^4^. Agricultural investments are highly challenging due to various financial and natural risks, including nutrient price volatility, market fluctuations, and supply chain disruptions^5^. This complexity underscores the necessity for precise, predictive models of crop production to support effective resource management, development of early warning systems, and enhancement of food security strategies.

Recent advances in remote sensing technology and numerical climate modeling have enabled the acquisition of detailed climate and soil data over broad geographic areas and diverse temporal intervals. By employing the Climate Model Intercomparison Project Phases 5 and 6 (CMIP5, CMIP6)^6,7^ simulations, researchers can project future climatic conditions up to 2100, significantly enriching climate-agriculture integrated studies^3,8,9^. Both machine learning and physical modeling, supported by remote sensing, have proven effective in addressing numerous agricultural challenges. For example, research has differentiated between irrigated and rainfed croplands under changing climate conditions^10–12^ and examined the effects of climate-induced droughts, hails, and floods on croplands^13–18^. Extensive use of satellite imagery has facilitated regional crop yield mapping and monitoring^19–22^, with numerous studies leveraging satellite-derived data for yield estimation^23–26^.

The challenges of crop production are primarily characterized by two factors: the expansion or degradation of arable land and the significant fluctuations in crop yields. Previous research has often overlooked the comprehensive interaction between these two critical aspects^27,28^. Our study addresses this oversight by introducing a combined methodology made of two major components. The first is a high-fidelity, data-driven approach to unearth historical correlations between climate variables and arable land dynamics. Based on the widely recognized CMIP5 climate models, we evaluate land utilization patterns. The second is a future yield changes forecasting based on climate conditions and fertilizer consumption, aiming to project shifts in agricultural production over a 7-year forecast period.

**Figure 1:**
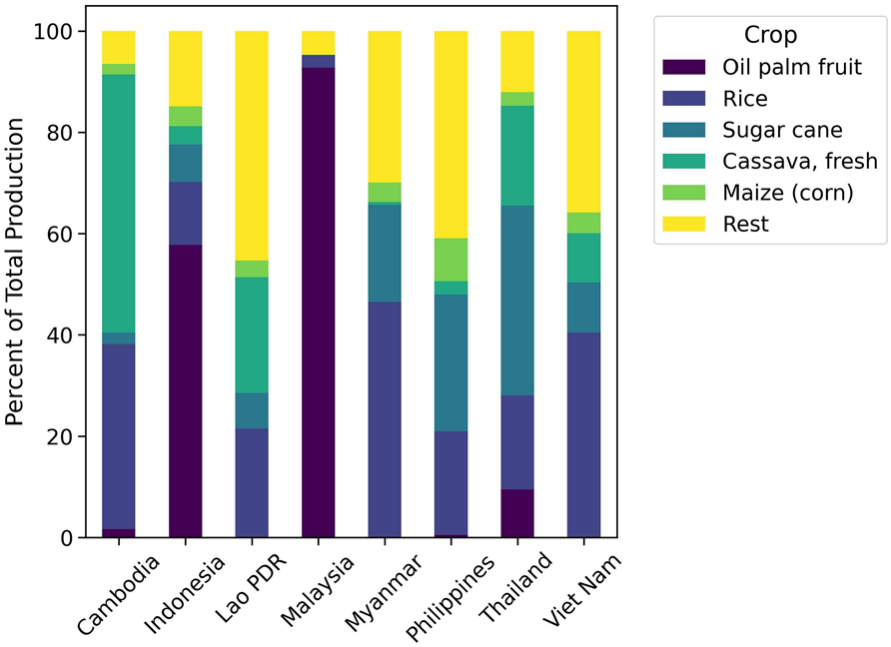
Percentage distribution of crop production across Southeast Asian countries, based on data from^31^ (This figure was created in Python 3.10 and the Jupyter Notebook programming interface).

**Figure 2:**
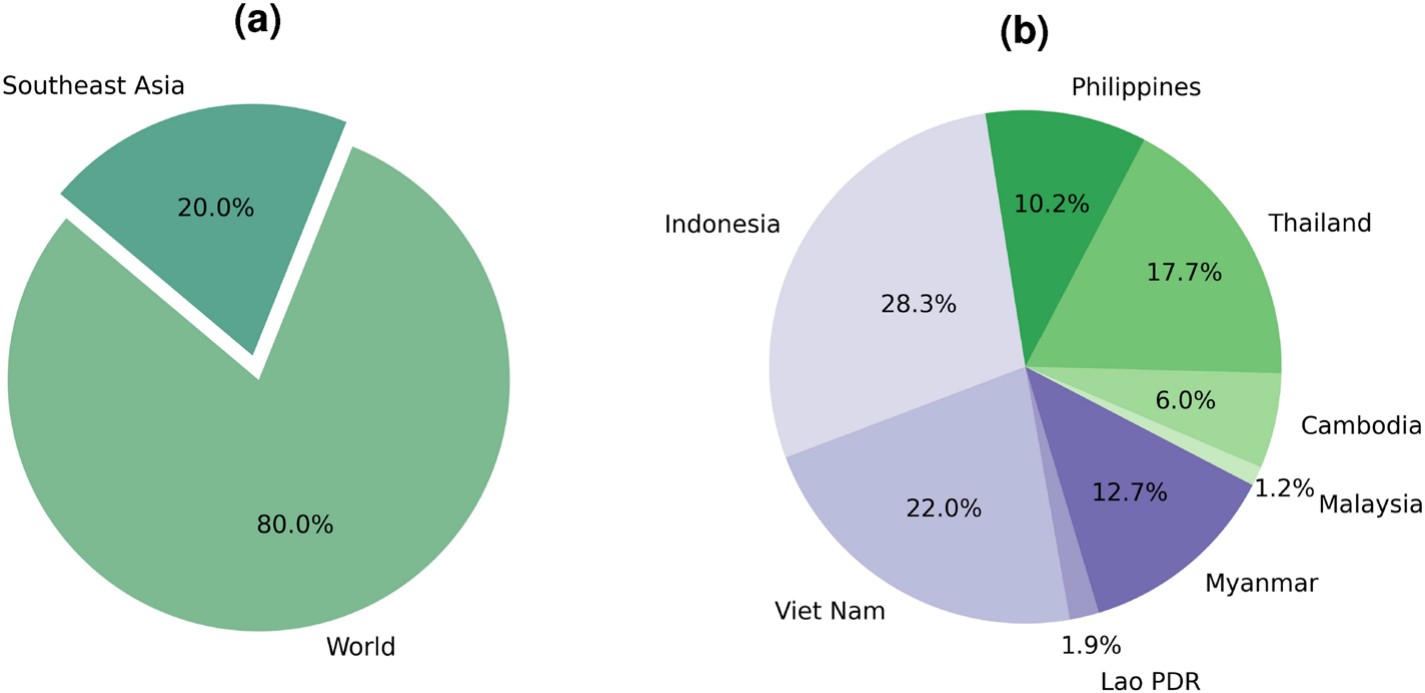
(**a**) Global Rice Production; (**b**) Rice Production in Southern Asian Countries in 2022^31^. (This figure was created in Python 3.10 and the Jupyter Notebook programming interface).

We have implemented and validated our combined approach across countries in Southeast Asia—Cambodia, Indonesia, Lao PDR, Malaysia, Myanmar, Philippines, Thailand, and Viet Nam - covering the period from 1966 to 2021. This region, known for its substantial agricultural potential, is highly vulnerable to climate change and extreme weather events, with a critical dependency on climatic conditions for its food supply chains^29^. Crop production in Southeast Asia is a significant part of the region’s economy and food security. Cambodia, Indonesia, Lao PDR, Malaysia, Myanmar, Philippines, Thailand, and Viet Nam produce a range of agricultural products, including palm oil, rice, maize, cassava, sugarcane, and others^30^. The detailed distribution of crop production based on Food and Agriculture Organization Corporate Statistical Database (FAOSTAT) data^31^ is illustrated in Fig. 1. From here, one can observe that rice is commonly the dominant production type for this region. Our study particularly focuses on rice production, reflecting its foundational role in the region’s agricultural landscape and its importance to food security^32^. The rice production distribution around the globe and within Southeast Asia is reflected in Fig. 2.

Despite notable progress in yield and crop modeling, many countries in the Asian region are understudied (except for India and China). Therefore, this gap presents an opportunity to develop regionally sensitive models that can account for region-specific factors and provide better quality results within the areas of interest. Thus, our study not only fills a critical research gap but also aids in sustainable agricultural development by enabling regional stakeholders with the tools needed for informed decision-making in long-term agricultural planning, investment, and economic policy development. This collaborative effort is essential for addressing the impacts of climate change and securing food resources in vulnerable areas.

## Results and discussion

**Figure 3:**
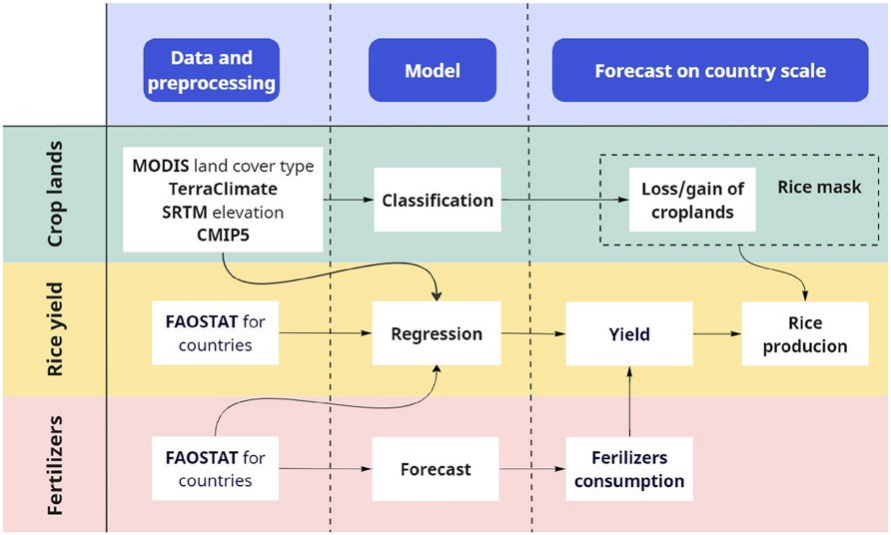
Research methodology. (This figure was created with Miro online whiteboard (no version provided) www.miro.com).

The agricultural productivity of croplands is influenced by a combination of climatic and anthropogenic factors, including temperature, precipitation, pesticide/herbicide application, pollution, fertilizer usage, pH regulation, tillage practices, and others^33–35^. Given the complexity and interdependence of these elements, our study adopts a holistic approach by focusing on the primary determinants of agricultural output: climate and fertilizers. By developing models to examine the cropland dynamics and project changes in fertilizer consumption, we aim to predict shifts in rice production, taking into account potential shifts in arable lands, yield variations and fertilizers usage. The methodology of our research is illustrated in Fig. 3, which also outlines the data sources we have utilized. Detailed information on the datasets can be found in the “Data and preprocessing” section.

**Figure 4:**
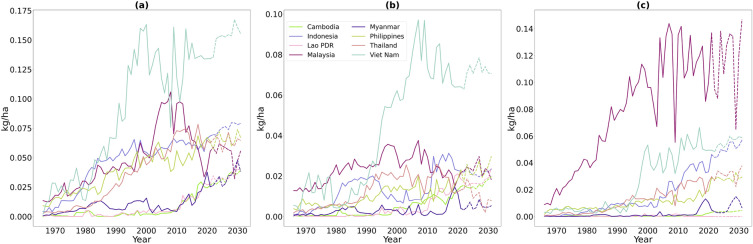
Historical and projected consumption of three primary fertilizers types: (**a**) Nitrogen, (**b**) Phosphorus, (**c**) Potassium. The solid line represents consumption data from 1966 to 2021, while the dashed line indicates projections from 2022 to 2031, measured per unit of arable area (This figure was created in Python 3.10 and the Jupyter Notebook programming interface).

Over the past 150 years, the application of fertilizers has significantly enhanced crop yields^36^. Figure 4 demonstrates the agricultural consumption of three major types of fertilizers in the years 1966–2021, along with projections for the next decade, based on our autoregression model (detailed in the “Rice yield model” section). The data reveal distinct consumption patterns for each country, reflecting varying fertilizer usage dynamics. These patterns suggest that appropriate fertilizer use could potentially mitigate the negative effects of climate change on crop production.

Our research aims to account for the influence of climate on cropland status, incorporating social factors for a more comprehensive analysis. In actual agricultural practice, lands suitable for crop cultivation are selected based on their potential, either as a conscious decision of farmers or processes analogously to natural selection, which identifies the most suitable combinations of land characteristics and other variables. This makes cropland suitability dependent on both social and climate conditions. Therefore, we consider past agricultural land as a predictive factor together with climate conditions to capture complex interactions between socioeconomic and climatic patterns.

### Cropland suitability

To model cropland suitability, we integrated climate and elevation data to develop a range of machine learning models, aiming to effectively incorporate social factors. These models range from conventional algorithms to more advanced neural network architectures, including Multilayer Perceptrons (MLP) and Convolutional Neural Networks (CNN). CNNs have garnered significant attention in climate and weather forecasting owing to the spatial nature of the data^25,37,38^. Recent research extends beyond these methods to consider recurrent neural networks and deep neural networks for capturing intricate spatio-temporal relationships in the data^19,39–41^ for regression problems in weather forecasting and climate modeling. Additionally, we used bioclimatic variables, which are climate indices updated annually, to analyze trends in temperature and precipitation. These variables offer insights into the current climate conditions (for more details, see “Data and preprocessing” section). These indexes are crucial for identifying and understanding climatic patterns.

In order to conduct a fair evaluation of the models, it is essential to select appropriate metrics for the task in question. Balanced accuracy is a performance metric that measures the percentage of correct predictions with respect to the share of each class, making it particularly useful when dealing with imbalanced classes where one class is underrepresented compared to the other. In this study, we use balanced accuracy to evaluate the performance of our classifier in distinguishing between the presence and absence of crops (denoted as class 1 and class 0, respectively). We estimate the precision (the tendency not to predict false croplands) and recall (the ability not to predict false non-croplands) using the optimal threshold based on maximizing the *F*-Measure—the harmonic mean of precision and recall. Among the models tested, the XGBClassifier^42^ outperforms its counterparts (see Table 1). While slightly behind CNN, CatBoost, and Random Forest, this model has the highest Balanced accuracy, Recall, and ROC-AUC values, indicating its superiority in cropland modeling based solely on climate conditions.

**Figure 5:**
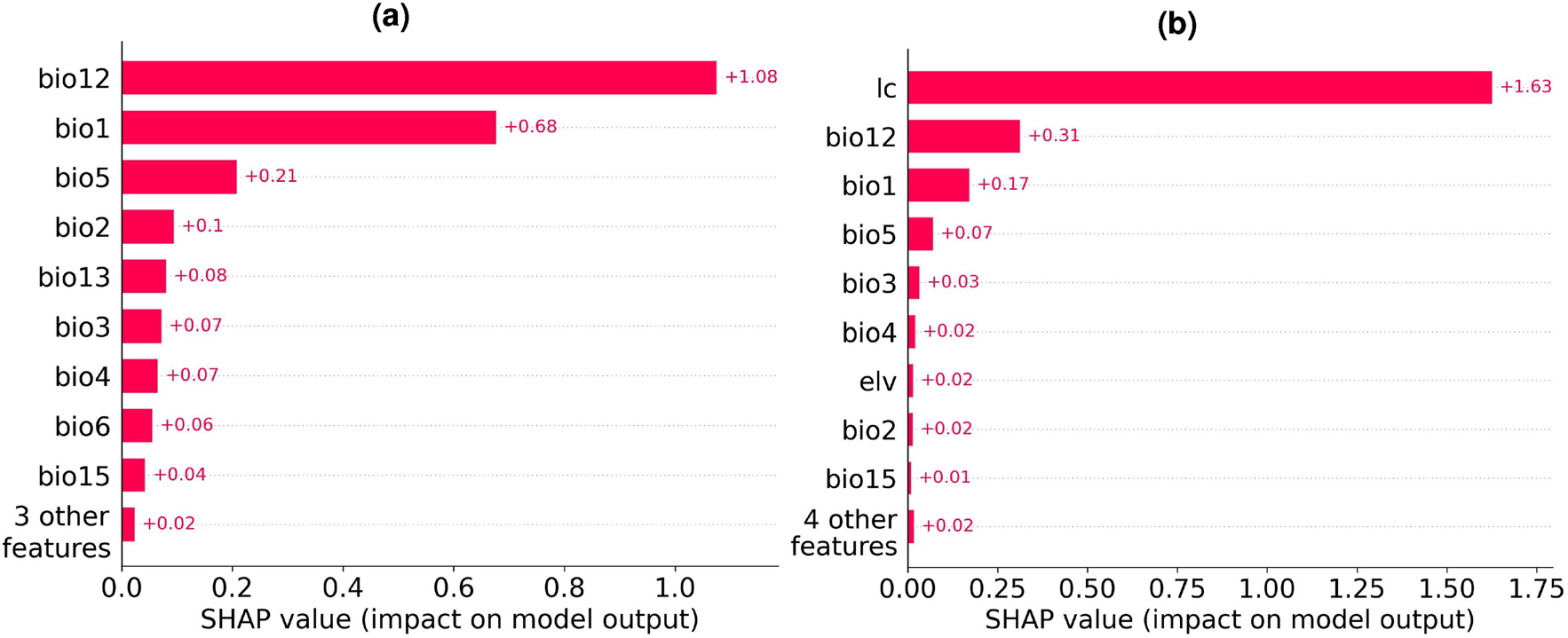
Feature importance evaluated using the SHAP tool for models with different feature sets. Features: lc—land class 7 years prior; bio1 to bio12—see notations in Table 5. (**a**) XGBClassifier via climate features, (**b**) XGBClassifier via climate features and previous land usage (This figure was created in Python 3.10 and the Jupyter Notebook programming interface).

Having identified the XGBClassifier as the top-performing model for cropland suitability classification, we trained it on both climate conditions and social factors. For the social factors, we considered land usage over the 7 years preceding the prediction date. This period reflects the socioeconomic influences on agricultural land use. A feature importance analysis conducted using the SHAP tool, which applies Shapley values from game theory to explain model outputs^43^, confirms that prior land use is the major factor contributing to the superior performance of the resulting model (Fig. 5).

**Table 1:** Classification metrics on test data. The standard deviation was computed based on a sample size of N = 10.

Model	Classification threshold	Balanced accuracy	Precision	Recall	ROC-AUC
Cropland suitability via climate conditions					
Logistic Regression^44^	0.15	0.750	0.296	0.610	0.871
Random Forest Classifier^45^	0.24	0.699	0.657	0.415	0.914
Naive Bayes^46^	0.98	0.733	0.376	0.534	0.891
MLP Classifier^47^	0.24	0.797	0.455	0.651	0.909
AdaBoost Classifier^48^	0.32	0.770	0.437	0.598	0.714
CatBoost Classifier^49^	0.32	0.802	0.660	0.624	0.959
XGBClassifier^42^	0.34	**0.813**	0.651	**0.653**	**0.960**
Convolutional Neural Network^50^	0.31	0.792	**0.669**	0.501	0.822
Cropland suitability via climate and socioeconomic conditions					
XGBClassifier	$0.49 \pm 1.8{\times }10^{-2}$	$0.959 \pm 1.3{\times }10^{-6}$	$0.939 \pm 3.2{\times }10^{-6}$	$0.924 \pm 2.8{\times }10^{-6}$	$0.992 \pm 5.3{\times }10^{-5}$

Bold values represent the highest metrics.

These findings highlight the importance of considering past agricultural land use when predicting the future status of croplands. This observation can be interpreted from various perspectives that are not mutually exclusive. First, it suggests that landowners may rely heavily on traditional farming methods instead of adapting new techniques that account for shifts in climate conditions. Second, it indicates that landowners might consider local factors not captured in our climatic and agricultural datasets. Third, landowners employing effective practices, whether based on empirical evidence or not, may benefit from a positive feedback loop, gaining better access to resources like fertilizers or financial assets. Overall, this feature highlights the critical socioeconomic conditions influencing cropland usage.

**Figure 6:**
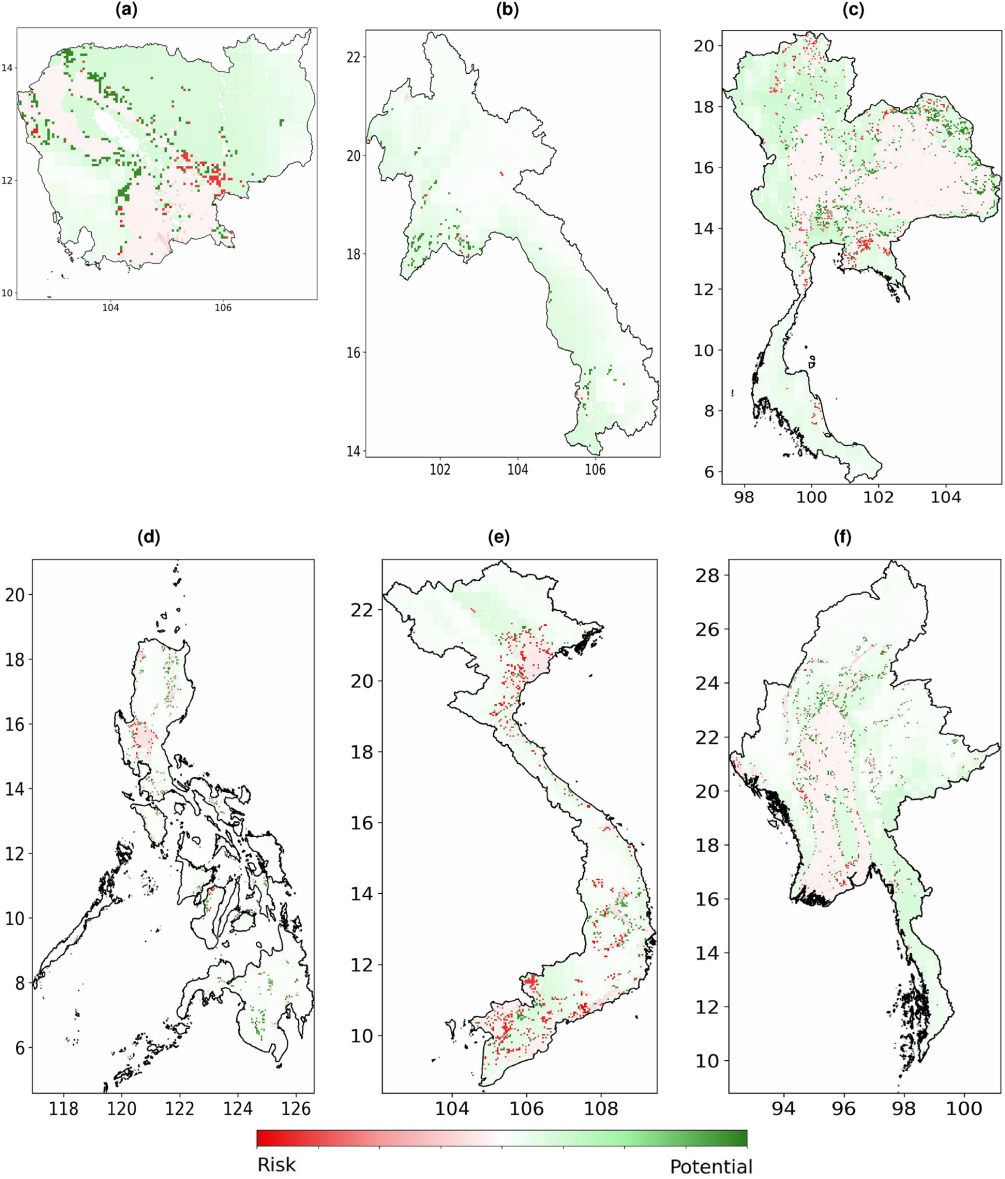
Projected changes in cropland area by 2028 relative to 2021, forecasted by the climate model for (**a**) Cambodia, (**b**) Lao PDR, (**c**) Thailand, (**d**) Philippines, (**e**) Viet Nam, (**f**) Myanmar. The horizontal axis represents longitude and the vertical axis latitude. (Maps were created using Rasterio version 1.3.9^51^ and Python version 3.10).

### Variations of land arability and rice production due to the climate change

Country-level agricultural rice production depends on the total area dedicated to arable lands and fertilizer use. Thus, we model potential changes in cropland area pixel-wise for Southeast Asia and fertilizer usage for each country.

Climate change leads to variations in cropland suitability, which we model using XGBClassifier, considering socioeconomic and climate change factors. Figure 6 shows the map with marked pixels having a high probability of crop status changes in the year 2028 compared to the year 2021, according to our model. Green color highlights the pixels with arable lands potential to expand, while red color—those with the potential to degrade.

In Cambodia, one can observe that the area near the Mekong River at the intersection of Kampong Cham, Kampong Thom, and Kratié provinces is to experience the most intense risk for arable lands in 2028, further, are to the South-East from the Tonle Sap Lake is under moderate risk. However, the North-Easten half of the country becomes potentially opportune for arability. Moreover, South-Western regions, such as areas near Kampong Chhnang city and Pursat province that are close to Lake Tonlé Sap and areas in the Aural District, West of the capital Phnom Penh, close to the Cardamom Mountains, are also potentially favorable for arability.

Considering Lao PDR, the proximities of the Mekong River near Nen Ngam Reservoir are projected to be more suitable for arability with minor exclusions. Overall, the southern and Western parts of the country demonstrate moderate potential for arability.

Khorat Plateau in Thailand is projected to be under moderate arability risk, along with areas near Ping River in the middle part of the country. Moreover, arable lands in the Chachoengsao district, on the border with Cambodia, and some lands in the North near Myanmar are at high risk. However, North-Easter lands near Lao PDR are demonstrating high potential prior to 2028.

In the Philippines, Mindanao islands are projected to have a favorable environment for arable lands. However, the central parts of Luzon islands are considered to be under moderate risk with minor high-risk fields.

Viet Nam’s arable lands are projected to be among the most at risk in Southeast Asia. Arable lands around Hanoi, South-West towards Thanh Hoa, and down the Ca river near Vinh city are under arability loss risk in 2028. Also, Tay Ninh, An Giang, Kien Giang provinces, Tan Hung and Vinh Hung districts are under high cropland suitability loss risks, together with areas near Song Ray Lake and areas South of Can Tho city. On the other hand, areas North of Hanoi and a few areas on the intersection of Phu Yen, Dak Lak, and Bihn Dihn provinces show some potential for new arable lands.

Finally, Myanmar has no significant loss of arable lands. It has moderately risky areas in Ayeyarwady, Bago, and Magway provinces and arability-gain areas dispersed among the Sagaing, Kachin, and Shan provinces. On the other hand, the vast majority of the country has moderate potential for arability gain in the Eastern and Southeast provinces.

Table 2 demonstrates the results of rice yield modeling utilizing the cropland suitability model and fertilizer usage. See section “Rice yield model” for the modeling details. Here, yield is estimated per modeling grid cell of $49 \text { km}^2$ area. A negative yield or production percentage value indicates a decrease, whereas a positive value represents an increase. The combined yield model shows $R^2 = 0.97$ and mean absolute percentage error $MAPE=4.2\%$ on test data.

Analyzing the results indicated in Table 2, we emphasize that under a negative scenario of no action to reach for utilizing potentially arable lands (Overall Croplands, bold column), Viet Nam, Philippines, Philippines, Lao PDR and Indonesia are expected to loss significant number of total arable lands, meanwhile Thailand, Myanmar and Cambodia are expected to have moderate losses. On the other hand, having reached for potential lands, all countries may not even mitigate severe losses but increase the total area of arable lands, except Indonesia (Overall Croplands, parenthesized column). A similar picture is for paddy rice fields only (Paddy Rice Croplands column).

Due to the climatic conditions in 2028 and forecasts of fertilizers usage (see Fig. 4), per area yield is considered to fall for each country, except for the Philippines. Note that for Cambodia, Myanmar, and Viet Nam, it is larger than $10\%$. Despite the fact that some countries like Myanmar, Thailand, and Cambodia are not to experience a dramatic drop in the total area of croplands and paddy rice fields, they are to experience a yield drop. This is caused by a drop in fertilizer usage for these countries; see Fig. 4. For Cambodia there is a low usage of Potassium, for Myanmar it is both Potassium and Phosphorus and for Thailand it is Nitrogen, Phosphorus and Potassium.

Next, we estimate the total Production Change for the countries’ areas in the last column. According to our Cropland Suitability XGBClassifier, in a negative course, i.e., no reach for potentially arable lands and loss of current ones, the overall picture is similar or more dramatic for all countries. However, if some countries, e.g., Lao PDR, Thailand, and Malaysia, reach for potentially arable lands, they might not just mitigate losses but even increase their production significantly.

To sum up, this study focuses on identifying potential risks rather than proposing development strategies. According to our findings, Cambodia and Viet Nam face severe threats in rice production, while the Philippines is expected to experience growth. Moreover, if countries take an opportunity to utilize potential paddy rice croplands, they might mitigate production drop risks and even increase their rice output. These findings highlight potential risks and emerging opportunities for policymakers that define agricultural strategy.

**Table 2:** Projected percentage changes in yield and rice production for 2028 compared to 2021.

Country	Cropland changes, %				Per area yield change,%	Production change, %	
	Overall croplands		Paddy rice croplands				
Cambodia	− **4.3**	(10.5)	− **1.1**	(5.3)	− 13.1	− **14.1**	(− 8.6)
Indonesia	− **17.3**	(− 1.7)	− **3.5**	(− 2.5)	− 5.9	− **9.1**	(− 8.2)
Lao PDR	− **9.2**	(1.7)	− **1.2**	(14.3)	− 5.8	− **7.2**	(7.4)
Malaysia	− **10.4**	(8.9)	− **0.9**	(0.9)	− 1.1	− **2.0**	(− 0.3)
Myanmar	− **3.7**	(5.8)	− **1.2**	(2.4)	− 21.4	− **22.5**	(− 19.6)
Philippines	− **11.4**	(10.6)	− **1.5**	(1.1)	6.8	**5.1**	(7.9)
Thailand	− **2.6**	(5.1)	− **0.7**	(10.1)	− 6.1	− **6.7**	(3.5)
Viet Nam	− **22.5**	(− 11.1)	− **9.3**	(− 7.2)	− 10.6	− **18.9**	(− 16.9)

Bolded values indicate changes attributed solely to arable land degradation. Values in parentheses reflect adjustments for both land degradation and land expansion.

### Comparative analysis with existing research

Comparing our study’s results with those of existing research, statistical analyses, and projections provides valuable insights. We categorize these comparisons into two main areas: machine/deep learning modeling of cropland suitability and rice-related projections.

In the field of rice yield projections, the IPCC Sixth Assessment report provides^52^ projections of agricultural production up to 2040 and 2080 for Southeast Asia countries. This report indicates similar results to those of our research for Thailand, Cambodia, and the Philippines, which support the validity of our research and align it with credible IPCC organizations. However, our research provides a more detailed analysis by countries and a closer projection timeline.

Moreover, research^53^ evaluates production potential, net exports, and yield gap projections by 2040 in Southeast Asia. The authors took into account current crop management methods via the results of questionnaires of agronomists collected by countries. Yield potential projection was conducted using ORYZA v3. This is a plant growth simulation software that is a valuable tool for understanding the genetics of a specific rice plant and improving local agricultural practices, but it is not built for country-scale analysis. Furthermore, their analysis of yield gaps and rice demand-supply relied on data from local reference weather stations. Authors in^54^ developed a probabilistic framework for predicting the Vegetation Health Index (VHI) up to 3 months in advance, using a Quantile Random Forest model that correlates VHI with rice price shocks^54^. Our approach extends this by linking predicted cropland suitability directly to rice yield, providing a more direct connection to cropland status than just economic indicators like price shocks.

Regarding machine and deep learning, studies^23,25^ propose neural network frameworks for predicting yields of rice, soybean, and corn, leveraging recurrent and convolutional architectures. Authors consider the USA corn belt and China, respectively, which are already well-studied areas. These studies focus on the performance and analysis of neural networks tested solely, without applying them further to obtain insightful results for practical applications.

In contrast to the studies and reports above, our study, first, allows for a more transparent and focused modeling methodology and tools. Specifically, by using CMIP5 climate projections, analysis of different predictor spaces—bioclimatic variables with or without socioeconomic factors and testing a wider range of learning models. Second, we build cropland suitability prediction and use it to project rice production up to 8 years ahead. Finally, our study includes risk analysis for rice production in Southeast Asia, which is understudied in the research field.

## Materials and methods

### Data and preprocessing

In this study, we develop a model employing several open datasets detailed in Table 3. We get remote sensing data with Google Earth Engine^55^, and we took MCD12Q1 Land Cover Type and TerraClimate datasets from this platform. We assume that the elevation is invariant through all the considered time. The land classification is based on the University of Maryland classification^56,57^. We transform the land cover to binary classification with crops (labeled as class 12 in the source) and non-crops (all other classes). Evaluation of the initial data revealed an imbalanced distribution of classes, with an average of 11% of all lands assigned to crops.

**Table 3:** Description of the datasets used in the study.

Dataset name	Variable	Time coverage	Spatial resolution	Temporal resolution
NASA SRTM Digital Elevation^58^	Elevation	–	$0.00028^\circ$	–
MCD12Q1 Land Cover Type^56^	LC_pe2	2001–2022	$\frac{1}{20}^\circ$	Yearly
TerraClimate^59^	Minimum and maximum temperatures, precipitation	1958–2023	$\frac{1}{24}^\circ$	Monthly
CMIP5^6^	Monthly mean of the daily-minimum and daily-maximum near-surface air temperatures, sum of precipitation at surface	1950–2100	$\frac{1}{2}^\circ -\frac{3}{2}^\circ$	Daily
NESEA-Rice10^60^	Paddy rice map	2017–2019	$0.0001^\circ$	Yearly
Global Administrative Areas^61^	Administrative boundaries of the countries	2022	–	–
Food and Agriculture Organization Corporate Statistical Database^31,62^	Rice (production quantity), rice (area harvested), fertilizers by nutrient (agricultural use)	1961–2021	–	Yearly

We utilized TerraClimate monthly means^59^ as historical climate data. We consider the future climate data from various CMIP5 simulations based on multiple evaluations conducted by different groups^63,64^ to ensure the high-fidelity and robustness of the results. Table 4 lists simulations employed in this study under the moderate Representative Concentration Pathway (RCP) 4.5 scenario of greenhouse gas concentration trajectory. To reach the consistent gridded data, we downscaled MCD12Q1 and CMIP5 data to the resolution of TerraClimate using nearest and bilinear interpolation methods, respectively. For NESEA-Rice10, we downsampled the data to the resolution of TerraClimate using the nearest resampling method. The grid coarsening and upscaling were implemented using Rasterio^51^. Application of these methods for up and down-scaling is a community-accepted approach in aligning multiple gridded data sources^65^.

**Table 4:** CMIP5 simulations used in this study.

Model name	Institution
CNRM-CM5	Centre National de Recherches Météorologiques, France
GFDL-CM3	NOAA Geophysical Fluid Dynamics Laboratory, United States
MPI-ESM-MR	Max Planck Institute for Meteorology, Germany

The climate data utilized have daily (CMIP5) and monthly (TerraClimate) temporal resolution. During the preprocessing stage, we calculate the mean maximum and minimum temperatures, as well as cumulative precipitation figures, for each month. Furthermore, historical and future climate data are used to calculate annual values of bioclimatic variables according to the approach developed in^66^. The Table 5 contains the list of variables. Bioclimatic variables are important for understanding how climate affects cropland usage. These predictors encompass various aspects of climate, including annual conditions such as mean temperature and precipitation, as well as seasonal variations like temperature and precipitation extremes.

**Table 5:** Bioclimatic variables.

Bioclimatic variable	Description
bio1	Annual mean temperature
bio2	Mean diurnal range
bio3	Isothermality
bio4	Temperature seasonality
bio5	Max temperature of warmest month
bio6	Min temperature of coldest month
bio7	Temperature annual range
bio12	Annual precipitation
bio13	Precipitation of wettest month
bio14	Precipitation of driest month
bio15	Precipitation seasonality

**Figure 7:**
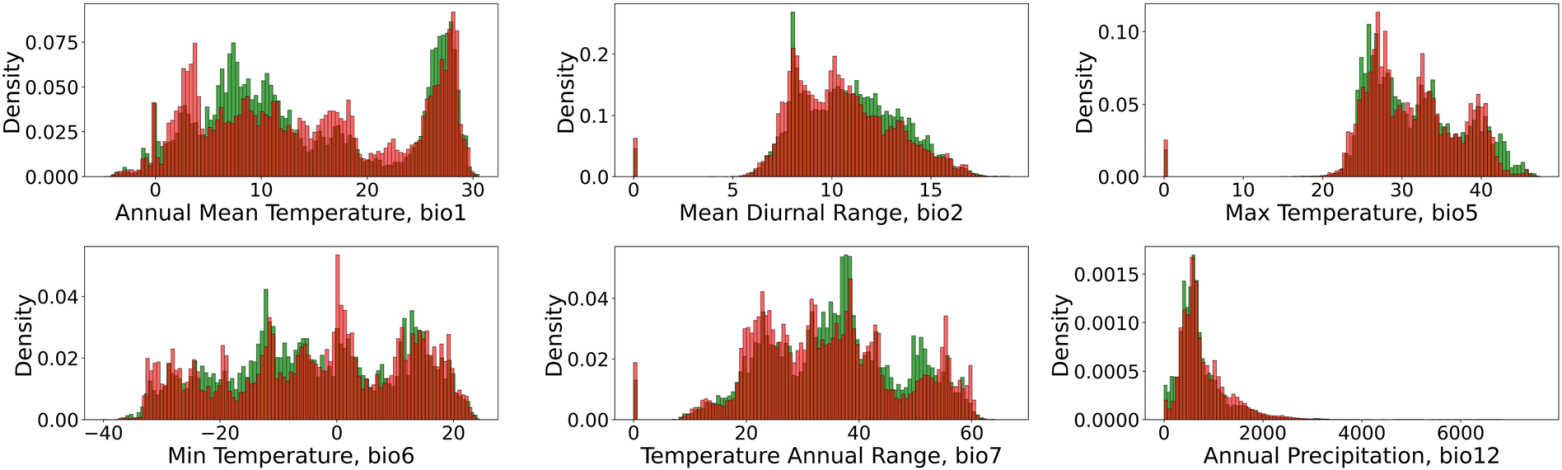
Climatic indices density distribution of samples lost (red) or acquired (green) cropland status. Temperature is measured in $^{\circ }$C, and precipitation is in mm (This figure was created in Python 3.10 and the Jupyter Notebook programming interface).

Figure 7 illustrates the density, i.e., the concentration of pixels for a specific variable bin, histograms of the distribution of bioclimatic indices for the pixels that either lost their crop production status (marked in red) or acquired it (marked in green) over the years modeled. The profiles of some features show a distinct shift, which is likely to aid in predicting the status of croplands. This shift indicates that relatively warm conditions, such as an average yearly temperature between 5 and $10^\circ$C and a minimum temperature of the coldest month between $-20$ and $-10^\circ$C, are likely to result in the emergence of croplands. In contrast, harsher conditions can lead to their disappearance.

In agricultural settings, climatic variables have a substantial impact on the growth and development of crops. These effects may manifest immediately, such as when a hailstorm or flood damages crops, or they may be delayed, such as when soil loses vital nutrients due to prolonged changes in precipitation or droughts. Our study aims to predict the future suitability status of arable lands. We assume a land transformation happens a year after actual climate conditions occur. Recent studies^12,67,68^ revealed that atmospheric climate conditions play a significant role in cropland suitability and crop yield. Thus, we include bioclimatic variables in the predictor’s list. However, arable land suitability is also influenced by socioeconomic factors and water availiability. In order to take into account the latter and former, we include the history of previous cropland usage. More precisely, we model cropland usage status for a specific year by incorporating bioclimatic variables a year before and cropland usage status 7 years prior. We then develop a machine learning tool that utilizes climate data and land usage history to make these predictions (see below). Classical performance metrics were used to assess the model’s performance using data bootstrapping. As our study primarily focuses on the potential decline in soil productivity, recall appears to be of greater significance. The significance of the delayed and immediate effects of climatic parameters on agricultural production also depends on other variables. Fertilizers, a major attribute of the Green Revolution, can help reduce the potential production loss caused by weather and generally increase land productivity. Based on agricultural statistics, one can identify trends in fertilizer consumption and predict future use.

The exact assessment of anticipated changes in land cover with respect to the impact of climate change on rice-growing fields is made possible by utilizing paddy rice maps. This data is available at high resolution in Southern Asia with the NESEA dataset^60^. Additionally, we make use of a wealth of national statistics from the Food and Agriculture Organization Corporate Statistical Database^31,62^.

### Cropland suitability via climate and socioeconomic conditions

At this stage, we want to demonstrate the relationship between the climate and socioeconomic conditions for a specific arable land suitability. The area of interest is considered as a uniform spatial grid, where each pixel has elevation value, bioclimatic values (derived from historical climate and future climate projections), indicator of being used and designated land class as the target label. With collected data, we train a binary classifier to predict the probability of assigning either class 1 (arable land) or class 0 (not arable land) to a specific sample, which is described with features listed in Table 5 along with elevation and 7 years prior this specific land usage. The classification threshold serves as a decision threshold that maps the classifier output probability of a sample being assigned to class 1 (presence of crops) to its actual binary category. We consider potential lands to become utilized, i.e., assigned to class 1, only when the cropland suitability classifier is highly certain and there were active arable lands in 7 kilometers of proximity in 2021. On the other hand, for risky arable lands, i.e., class 0, we considered only the classifier’s certainty. Overall, this model utilizes TerraClimate and land covers class mask—MCD12Q1. The former dataset covers the 1958–2023 year range, meanwhile the latter covers 2001–2021, thus leveraging a 20-year period for training.

Extreme Gradient Boosting Classifier XGBClassifier^42^ was chosen as a machine learning backbone since it performs better than other tools when applied to the same data in our pilot study (Table 1, also see^69^). In the first step, all the features are used for training with grid search and StratifiedKFold cross-validation among several regularizations and decision tree parameters. The procedure of choosing optimal parameters is given in section “Classifier parameters”.

The alteration of arable lands may pose challenges in interpreting its impact on food security, particularly due to the lack of information regarding the specific crop types being cultivated in various areas, except rice. Available paddy rice dataset^60^ offers an opportunity to improve the precision of land assessment for rice forecast purposes. When using it as a mask for crop fields, we refer to this as the ”rice mask” and demonstrate the significance of utilizing these data in current research. By employing this mask, we enhance the accuracy of climate change impact evaluation on rice production compared to the general analysis that does not consider the specific location of rice fields.

### Fertilizer model

Fertilizer data was obtained from FAOSTAT^62^ as indicated in Table 3 and include the agricultural use of nitrogen *N* (in various chemical forms), potash $K_2O$, and phosphate $P_2O_5$. Fertilizer consumption data is available for the period of 1966–2021. Figure 4 illustrates their historical agricultural use *F* with solid lines. The forecast for the future year *y* is generated for country *c* and for each fertilizer with autoregression model and shown in the same plot with dash lines. We employed the Seasonal Auto-Regressive Integrated Moving Average (SARIMA) model for the forecast. This model takes into account the time series values in the past, modeling temporal dependencies in observation noise and considering seasonal dependencies for differentials of the original time series. See, e.g., Chapter 10 in^70^, for details.

### Rice yield model

To assess the potential effects of crop yield degradation, we build the regression model that learns the connection between climate, consumption of fertilizers time trends, and yield (per unit area) as the target variable following the methodology presented in^27^. We develop this approach to capture the link between socioeconomic traits and climate conditions. Climate features of the yield model include values of minimum and maximum for temperatures and precipitations, calculated as monthly means as well as variances of these values in the monthly distribution. We use climate data collected from the TerraClimate source within national borders that were acquired from the Global Administrative Areas dataset (see Table 3). This approach yields 72 features in total. Similarly to the climate model, we explore the ensemble of models listed in Table 4 to overcome potential biases of single CMIP projection for 2028 country-wise yield estimation. Overall, the rice yield model trains on data in the range of 1966–2021 and is limited by fertilizer data availability (1966–2021) and the TerraClimate range (1958–2023).

We utilize the specific year values *F* for nitrogen *N*, potash $K_2O$, and phosphate $P_2O_5$ to serve as three features in the modeling of yield. Mathematically, we set the functional dependence and estimate the unknown coefficients as follows:

1 $$\begin{aligned} \begin{aligned} Y_{cy} = M\left( pr_{cmy}, pr^{var}_{cmy}, tmax_{cmy}, tmax^{var}_{cmy}, tmin_{cmy}, tmin^{var}_{cmy}, F^N_{cy}, F^{P_2O_5}_{cy}, F^{K_2O}_{cy}\right) \end{aligned} \end{aligned}$$

where

- *Y* is rice yield,
- *c*, *m*, *y* represent country, month and year respectively,
- *pr* and $pr^{var}$ are precipitation level and its variance,
- $t_{\max }$ and $t_{\max }^{var}$ are maximum temperature and its variance,
- $t_{\min }$ and $t_{\min }^{var}$are minimum temperature and its variance,
- *F* are fertilizer consumptions,
- *M* is the XGBRegressor with number of ensemble members of 100, maximum tree depth of 2, the other parameters were set default.

To determine the yield as a target variable, we divide the rice production of a specific country by its corresponding cultivation area, with both values sourced from the FAOSTAT data (see Table 3). National statistics and climate data are utilized to obtain the necessary information for calculating yield forecasts. We then apply this regression model to estimate future rice yields in a given country. When combined with the expected reduction in area, it effectively predicts rice production.

### Relative yield change

We analyze the relative yield change caused by the effects of varying fertilizer usage and potential losses or gains of land area on the country level in Southeast Asia.

During this analysis, we estimated potential gains and losses of arable lands using the cropland suitability XGBClassifier model, projections of fertilizer usage by SARIMA, and a combined XGBRegression model for rice yield change. Next, we compared current arable lands distribution by pixels with the results of potential and risky arable lands by cropland suitability model to estimate potential overall loss or gain in arable land area for each country in Southeast Asia. Then, by comparing the rice mask with the results of the XGBClassifier, we estimated the percentage of area loss or gain solely for rice fields. Finally, we combined the per-area yield model with the arable land area percentage of change to get the production change.

## Numerical experiments

### Data analysis

Our study focuses on the proposed approach and its application in Southern Asia. We cover a diverse range of countries with varying levels of social and economic development, including Cambodia, Indonesia, Lao PDR, Malaysia, Myanmar, Philippines, Thailand, and Viet Nam. The region of the study is limited to a latitude range of $11^\circ$S to $60^\circ$N and a longitude range of $46^\circ$E to $146^\circ$E. The spatial resolution utilized is $1^\circ /24$ , which was determined through the algorithm described in section “Data and preprocessing”.

### Classifier parameters

We performed a grid search in order to estimate optimal hyperparameters for further modeling. Table 6 displays the initial parameter sets and the optimal values that we chose.

**Table 6:** Grid search ranges for hyperparameters.

Parameter	Tested set	Chosen value
$reg\_alpha$	0, 0.1, 1	0.1
$reg\_lambda$	1, 10, 100	1
$max\_depth$	3, 4, 5	5
$learning\_rate$	0.001, 0.01, 0.1	0.01
$n\_estimators$	50, 100, 200, 400	200

Figure 7 shows the distribution of most essential features. Aside from climate data and land class, the models listed in Table 7 include elevation (elv) and land class 7 years prior (lc). The inclusion of “memory” within the name indicates that historical land classes, i.e., prior land usage of this land, were also used as a part of its feature space.

**Table 7:** Models with features included. Bioclimatic variables are listed in Table 5.

Model name	Features
Climate model	11 bioclimatic variablies, elv
Climate model with memory	11 bioclimatic variablies, elv, lc

### Training and testing

The training and testing subset is acquired using TerraClimate data for the period specified in Table 8. To avoid any potential data leakage, we take great care in selecting the train and test data. Specifically, we ensure that the land class in any given year is never used as both a label for training and a feature for testing. The collected data for these years boast complete coverage within our area of interest, allowing for a comprehensive analysis.

**Table 8:** Modeling year ranges.

Model name	Model type	Train	Test
Climate model	XGBClassifier	2001–2019	2020–2021
Fertilizer model	SARIMA-X	1966–2019	2020–2021
Yield model	XGBRegression	1966–2019	2020–2021

**Figure 8:**
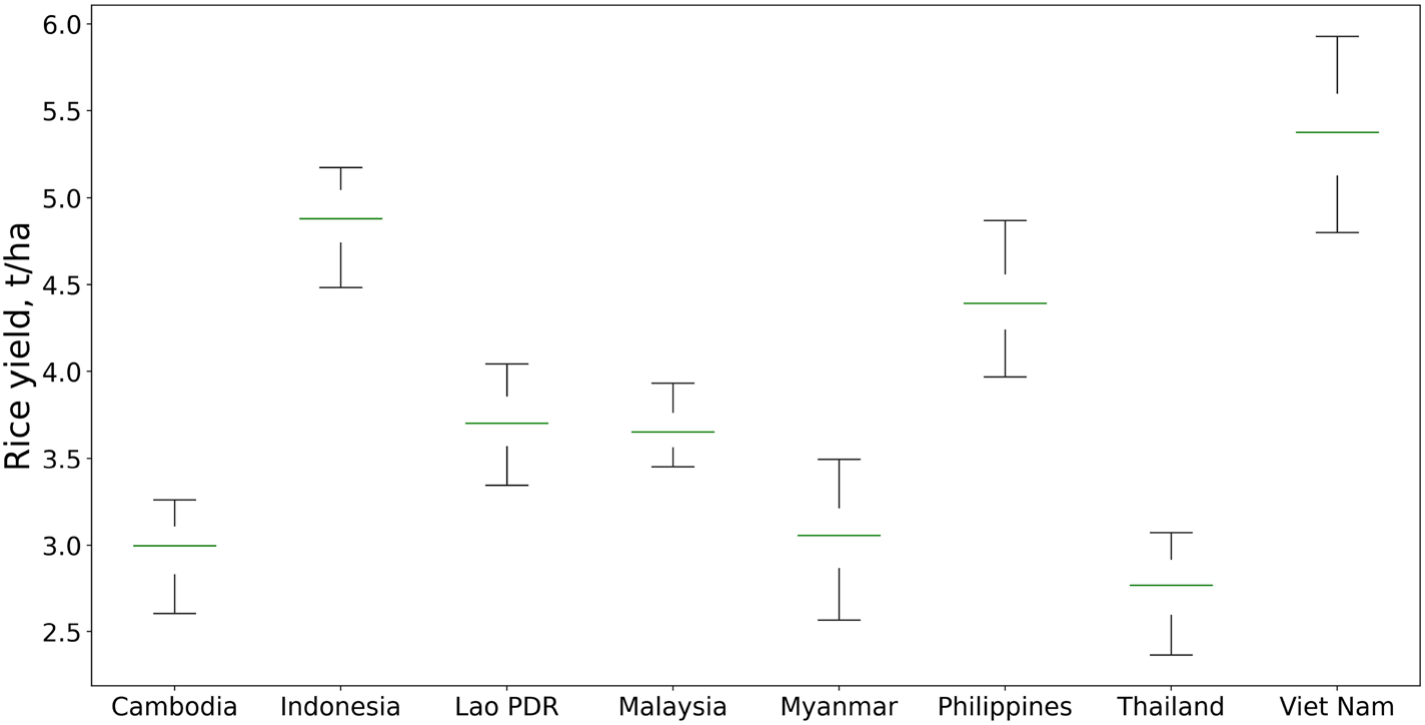
The uncertainty estimation for the yield model in 2028 with a 90% confidence interval, distribution means indicated by the green lines. The sample size for the analysis is N = 500 (This figure was created in Python 3.10 and the Jupyter Notebook programming interface).

The fitted model uses CMIP5 climate projections to make a forecast. Phase 5 is chosen since it has a better correspondence in temperature with recently observed data^71^. We assume that the suitability of climate models may vary depending on the chosen climate zone. To improve consistency, we create an ensemble projection by averaging the CMIP5 simulations listed in Table 4. This approach is widely employed^72,73^. Finally, we tested fertilizer forecasting modeling together with the yield model. Specifically, we trained the fertilizer forecaster and yield model in the same time range from 1966 to 2019. Then, we fed forecasts of the fertilizer model into the yield model to make a prediction of the testing range from 2020 to 2021. The quality of the latter was estimated to be $R^2 = 0.97$ and $MAPE=4.2\%$. To address the uncertainty of modeling, we implement a bootstrap procedure in both our climate and yield models. This enables us to assess the level of certainty associated with our estimations. Table 1 presents the variability of binary classification metrics for the cropland suitability classifier. We estimate the model uncertainty by building bootstrap confidence intervals for our model^74,75^. Figure 8 illustrates the distribution of projected rice yields for the countries being studied, with a 90% confidence interval.

### Constraints of the study

The primary constraint of this study pertains to the grid roughness. The spatial resolution employed (roughly 4500 m in cell length) is larger than the field size, resulting in several diverse areas within the same pixel. Additionally, this grid is uniform and does not correspond to the actual shapes of the fields. Lastly, our modeling relies on the datasets listed in Table 3. Some of these are the results of modeling studies, which inherently approximate natural phenomena and, therefore, are imprecise. Specifically, MCD12Q1 is a model product, meaning that the cropland maps are not ideal in classification. The CMIP5 projection that we used provides average climate evolution under the assumption of the RCP 4.5 scenario, which should be treated cautiously, taking into account that it is not an exact climate forecast. It influences our model and should be considered when interpreting the model outcomes.

Another limitation of this study is its focus on atmospheric variables without considering soil-related ones. This decision was based on the assumption of strong correlations between atmospheric and soil variables^76^. However, it is important to note that both atmospheric and soil variables are crucial factors in determining groundwater resources. Depletion of groundwater can lead to issues such as salinity hazards, which can adversely affect soil fertility and crop suitability^77,78^. Additionally, while our study indirectly models water availability through climate variables like precipitation, it does not directly investigate the availability and threats posed by different water sources, such as the distinction between surface water and groundwater irrigation. Despite Southeast Asia’s stable and low water stress index (see^79^, Chapter 1.7), incorporating irrigation patterns into modeling could provide additional insights for policymakers. Future studies should aim to include direct measures of water availability and quality to fully understand their impacts on crop productivity and land suitability.

Various studies conducted under the CMIP5/CMIP6 project can assist in overcoming the limitations of mathematical simulation in reproducing natural processes. Global-scale processes are incredibly complex. Accurate reproduction of such processes with mathematical simulations is still impossible. Each model has advantages and disadvantages in replicating changes occurring on land, in the atmosphere, in permafrost, or above the ocean. The appropriate work direction could be collecting the region-specific CMIP models of reasonable quality into an ensemble^80^. Addressing the above-mentioned drawbacks improves the accuracy of this study.

## Conclusion

This work presents evidence of the impact of climate on croplands and rice production in Southeast Asia. The study utilized a machine learning model that gathered bioclimatic variables based on historical climate data, socioeconomic factors, and fertilizer usage. These climatic indices, such as annual mean temperature, maximum temperature, and annual precipitation, were used to predict the presence or absence of cropland in the future based on climate projections. The paper contributes by proposing the framework for projecting rice production in countries. Firstly, it combines $7 \times 7$ km cropland suitability classifier, that takes into account both climate conditions and socioeconomic factors via land usage history. Secondly, it projects fertilizers usage that are the cornerstone of contemporary agriculture. Thirdly, it proposes a combined model that projects rice yield country-wise. Finally, analyses the risks and potentials in cropland arability with fertilizers usage to get relative changes in rice production in 2028.

The results showed that even moderate modeling suggests a high likelihood of severe conditions for growing crops in Cambodia, Myanmar, and Viet Nam. Consequently, these lands will either undergo a land transformation or experience a notable drop in rice yield. Additionally, the results indicate that a reach for utilization of potentially suitable croplands might not just mitigate the production drop risks but even be a path to prosper in rice production. Furthermore, the study allows for comparing neighboring regions. Underrated clusters were identified where crop potential is high, but the share of cultivated fields is low. This finding calls for local policy changes and investor initiatives, which could be used for regional development planning, creating agricultural road maps, water management, and more.

In addition to business motivations, the topic has a more comprehensive scope as it relates to global food security. Climate change is responsible for rearranging conventional food supply chains on regional and international scales. Predictions based on the findings of this study can help take measures to mitigate the impact of climate change on food security before actual transformations occur.

## Data Availability

All data references, links for loading the dataset used (limited years range), and source code needed to evaluate the conclusions in the paper are publicly available through Zenodo at https://doi.org/10.5281/zenodo.7960780.
